# The Abortive Treatment of Gonorrhœa in the Male by Injection of Clay-Earth

**Published:** 1874-12

**Authors:** Frederick William Godon

**Affiliations:** Late Interne, Rotunda Lying-in Hospital, Dublin, Ireland


### THE ABORTIVE TREATMENT OF GONORRHŒA IN THE MALE BY INJECTIONS OF CLAY-EARTH.

BY FREDERICK WILLIAM GODON, A.B., M.D.

*Late Interne, Rotunda Lying-in Hospital, Dublin, Ireland.*

On consideration of the severity and the duration of gonorrhœa as a disease, the complications to which it sometimes gives rise, the impairment of health, and the loss of strength which it entails, the surgeon is assuredly justified in making the attempt to cut short the disease at the beginning, and I beg leave to present a series of thirty-five cases of acute gonorrhœa treated most successfully by the injection of clay-earth. The clay is mixed with water to a consistency sufficient to allow it to pass through an ordinary rubber-bulb urethral syringe, by which means it is injected into the patient's urethra in the ordinary manner. Inasmuch as the earth is the essential of the injection, it should be mixed as thickly as possible in order to be injected into the urethra thoroughly and plentifully, and as the clay is non-irritating and antiseptic, it invariably produces a cooling and grateful sensation. When, however, the seat of the disease has extended itself so far along the urethra as to be beyond the reach of an injection, I have been in the habit of using a double catheter and douche, invented by my friend, Dr. Addinell Hewson, of Philadelphia, Pa., and described by him in Vol. II. Reports of the Pennsylvania Hospital, 1869. As such different opinions have been expressed by high authorities on the use of injections, some claiming them to be a fruitful source of stricture, and others perfectly innocent, admitting only a certain amount of plausibility to the first theory, I would claim that injections, used with judgment and under the proper indications, have been proved the most reliable and the best agent in our hands for the cure of this disease.

For any structural change to take place in the tunics of the urethra there must have existed some preceding inflammatory process; the more chronic the existence of such morbid process, the more likely is stricture to result. Inasmuch as injections are curative of inflammation, in just so much will they prevent this occurrence. The oldest of my present series of cases that I present now, dates back but three years, but I have seen the patient constantly during this time, and have attended him in two attacks of illness, and when I questioned him some two months ago in regard to his urethra, he told me he never had had his attention called to it since he was last cured of the clap, now three years ago. This gentleman is married and the father of a healthy boy. On examination of the urethra, which he kindly permitted in the presence of my colleague, Dr. C. E. Lockwood, of New York, with a bougie-à-boule as large as the meatus would easily admit, we could find no evidence of any stricture whatever.

In another of my cases, that of an attorney-at-law practicing in New York, and who came to me, October, 1873, to be treated for what he told me was his tenth attack of clap, the earth acted almost like magic, causing all pain on micturition to immediately cease, and on the third day after beginning treatment, he came to me and told me he considered himself entirely cured, and asked me to give him the same prescription for a friend who had been suffering from the same trouble for four months, and although under treatment during that time, was still unrelieved. In all my cases I began by ordering the patient enough pil. hydrarg., followed on the next morning by a saline, strongly recommending mag. cal. ʒ ij. mixed up with lemon-juice and water, to open the bowels freely. I made the first injection myself with what is called in New York a "PP" syringe, injecting certainly two drachms and a half of the mixture, and retaining it in the urethra for at least sixty seconds, and inducing the patient to wear a suspensory bandage, and to take as little exercise as possible. I have not, however, denied him red wine at his meals, if he has been in the habit of drinking it, nor have I ordered him to give up his after-dinner coffee. All late suppers, however, and alcoholic drinks I have prohibited, as well as any excitement calculated to produce sexual sensations. The mixture of earth and water I have told the patient to use at least every four hours, allowing it to remain in the urethra at least a minute, and to inject always after first passing his water. I assume that every surgeon is aware of the fact that nearly every patient who presents himself with gonorrhœa will account for his disease by over-exertion in the performance of the sexual act, and be very loth to admit that his companion was in anything but perfect health, and how he became affected in the manner in which he presents himself is entirely beyond his comprehension. Again, in most cases the patient is all anxiety to be well in a week's time at furthermost.

He never drinks, and the doctor can rest assured his directions will be carefully and strictly carried out to the letter. As the treatment of gonorrhœa is one which surgeons are so frequently called upon to treat, and as it never, even in its most acute form, confines the patient to bed, and puts the patient under the surgeon's control, he can never be assured that the patient is following directions, or taking his medicines, or using his syringe properly and faithfully. It is well known that the duration of the disease is very variable, even under the treatment of the best practitioners. I am induced to offer the following mode of treatment as one which, in my hands, has proved most effective to both my patients and myself. I am well aware, from conversation with a number of my personal friends in the profession, that each one has some favorite remedy for which he claims excellent results. Again, many still adhere to the older mode of treatment, and give copaiba and cubebs in some form, their doses varying greatly in quantity and frequency. Some excellent authorities treat the disease successfully by internal medication alone, while others combine the local with general treatment; a third class of practitioners only use and recommend the local treatment. In the cases cited I have used the local treatment only, giving no internal medication whatever, with the exception of the first cathartic before applying the earth to the seat of disease. My success in these cases, which I now present, leads me to suppose that they will prove interesting to those at all concerned in the treatment of this troublesome and constantly-occurring disease.—*American Journal of Syphilography and Dermatology.*

[Thirty-five cases are given which illustrates the value of the treatment.—EDS.]

